# The Impact of the Addition of a Virtual Reality Trainer on Skill Retention of Tourniquet Application for Hemorrhage Control Among Emergency Medical Technician Students: A Pilot Study

**DOI:** 10.7759/cureus.34320

**Published:** 2023-01-28

**Authors:** Adeel Arif, Rachel C Santana Felipes, Mia Hoxhaj, Michael B Light, Nicholas B Dadario, Brennan Cook, Matthew J Cataldo, Farrukh N Jafri

**Affiliations:** 1 Department of Emergency Medicine, White Plains Hospital, White Plains, USA; 2 Department of Medicine, Robert Wood Johnson Medical School, Rutgers University, New Brunswick, USA; 3 Department of Emergency Medical Services, Westchester Community College, Valhalla, USA

**Keywords:** vr tourniquet placement, tourniquet placement skill retention, tourniquet use, medical education and simulation, emergency medical technician, emergency medicine and trauma, skills and simulation training

## Abstract

Introduction: Trauma is a leading cause of preventable death in the United States. Emergency Medical Technicians (EMTs) often arrive first at the scene of traumatic injuries to perform life-saving skills such as tourniquet placement. While current EMT courses teach and test tourniquet application, studies have shown efficacy and retention of EMT skills such as tourniquet placement decay over time, with educational interventions needed to improve retention of skills.

Methods: A prospective randomized pilot study was conducted to determine differences in retention of tourniquet placement among 40 EMT students after initial training. Participants were randomly assigned to either a virtual reality (VR) intervention or a control group. The VR group received instruction from a refresher VR program 35 days after initial training as a supplement to their EMT course. Both the VR and control participants’ tourniquet skills were assessed 70 days after initial training by blinded instructors.

Results: There was no significant difference in correct tourniquet placement between both groups (Control, 63% vs Intervention, 57%, p = 0.57). It was found that 9/21 participants (43%) in the VR intervention group failed to correctly apply the tourniquet while 7/19 of the control participants (37%) failed in tourniquet application. Additionally, the VR group was more likely to fail the tourniquet application due to improper tightening than the control group during the final assessment (p = 0.04).

Conclusion: In this pilot study, using a VR headset in conjunction with in-person training did not improve the efficacy and retention of tourniquet placement skills. Participants who received the VR intervention were more likely to have errors relating to haptics, rather than procedure-related errors.

## Introduction

In the United States, trauma is the leading cause of death in individuals between the ages of 1 and 45. At the same time, uncontrolled hemorrhage has been identified as being the leading cause of preventable injury death [1-3]. As a result, there has been an emphasis on hemorrhage control training for lay people and first responders through programs such as the American College of Surgeons (ACS), the Stop the Bleed (STB) program, and Emergency Medical Technician (EMT) certification courses. Studies have found that prehospital tourniquet use is associated with reduced mortality in patients with vascular injuries [4,5]. While increased tourniquet use has shown favorable improvement in survival, inaccurate application or misuse may be ineffectual or exacerbate complications [5]. Therefore, tourniquet use is an essential skill for first responders. Current EMT certification requires individuals to identify life-threatening wounds and learn bleeding control techniques such as tourniquet placement [6].

Despite required certifications, current literature demonstrates poor retention of tourniquet placement skills along with a need for interventions to “refresh” these skills as soon as three months post-training [7,8]. Virtual reality (VR) has been used in educational settings in healthcare to simulate the benefits of in-person training, such as immersion and feedback while creating a sequence-driven learning experience that allows for repetitive practice of skills [9]. VR may facilitate asynchronous deliberate practice to improve the efficacy of sequence-driven medical procedures [10]. Further studies show that VR medical training can improve the accuracy and speed of medical tasks for medical students, including laparoscopic surgery and cardiopulmonary resuscitation (CPR) training [10-12].

The current research team has investigated different educational methods to improve tourniquet retention including the use of high-technology trainers [7] and mobile devices [8]. Additionally, the team has experience in VR development and investigation, having demonstrated a decrease in errors amongst residents in donning and doffing through a VR-based personal protective equipment (PPE) program [9]. Through these observations, the following question was asked: “Can an immersive VR experience, as an adjunct to in-person training, decrease errors in tourniquet placement on retention testing amongst EMT students?”. The primary objective of this study was to determine whether adding a tourniquet placement VR refresher to an existing EMT course would improve the efficacy and retention of tourniquet placement skills among EMT students. Secondary objectives included studying (a) if VR refresher training can decrease the total number of errors made during tourniquet placement and (b) determining the student's perceptions, comfort, and feedback of using VR as a modality to learn tourniquet placement skills. The team hypothesized that adding VR as a refresher course would result in improved retention of skills, reduced errors, and improved comfort in tourniquet placement compared to standard training.

## Materials and methods

This study was a single-blinded, prospective randomized education pilot study at a suburban, public community college with a prospective open-blinded endpoint design [13]. The Consolidated Standards of Reporting Trials [14] were followed. The study was approved by the Westchester Community College Institutional Review Board (IRB); verbal and signed consent was obtained from all participants.

Study participants and instructors

The participants were recruited from a local community college who were actively taking an EMT-Basic certification course. Three EMT classes were divided into control and intervention groups during the course of the semester. Traditionally, hemorrhage control was taught early in the semester, and students were able to practice during open lab sessions the week before their final examinations. During their initial assigned laboratory session, participants were debriefed regarding the purpose of the study and were able to withdraw at any time. Inclusion criteria required participants to be over the age of 18 and currently enrolled in the EMT course. Exclusion criteria included non-EMT students in the course and all instructors. Educators of the hemorrhage control techniques were instructors of the EMT class.

Each EMT-Basic class is a New York State (NYS) Department of Health (DOH) approved course consisting of 170 hours of didactic, psychomotor, and clinical instruction in compliance with the EMT-Basic National Standard Curriculum, outlined by the National Highway Traffic Safety Administration. The course is overseen by an NYS DOH Certified Instructor Coordinator with NYS DOH Certified Lab Instructors. At the conclusion of the class, students are eligible to take the NYS DOH EMT Practical Skills Exam and the NYS DOH EMT written exam, with successful completion resulting in certification as an NYS EMT.

Study protocol

**Figure 1:**
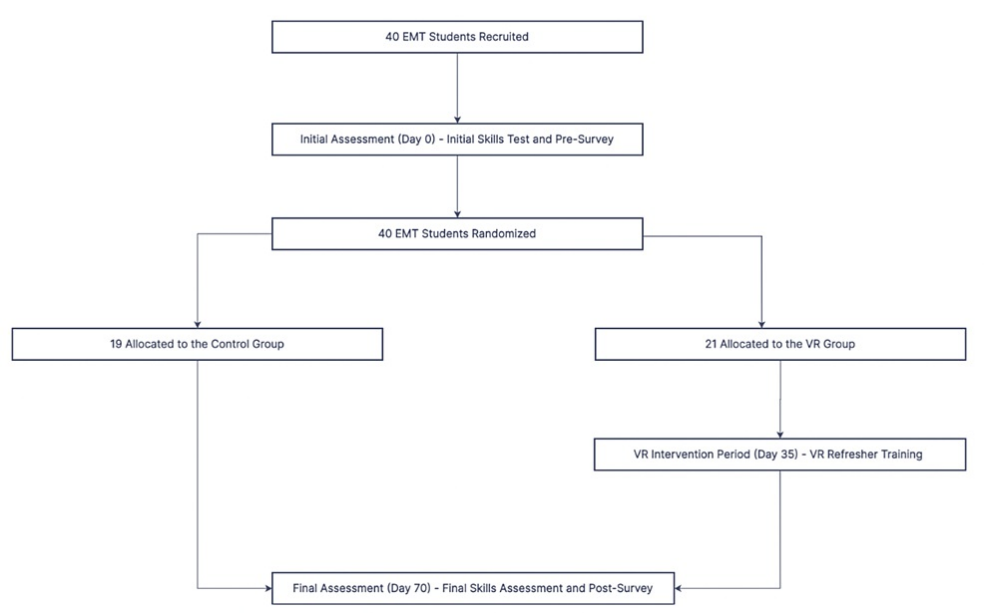
Study flowchart of control (no VR) and intervention (VR) groups VR: Virtual Reality, EMT: Emergency Medical Technician

This study ran from September 2021 to December 2021. Within this period, the study was divided into three sessions: Initial Assessment (Day 0), VR Intervention Period (Day 35), and Final Assessment (Day 70) (Figure 1). Upon enrollment, participants were assigned an identification study number and were randomized into one of two study arms: VR or Control, using computer-generated software. Participants remained unaware of their group classification until day 35 on which the intervention was conducted. Tourniquet skill raters remained blinded to the participants' classification throughout the duration of the study. Additionally, grading rubrics were validated through rater calibration before day 0. The final assessment was performed on day 70, just before the previously scheduled open-lab session, to avoid confounding with the independent practice for the final examination.

Initial assessment

Once participants were debriefed and completed their consent forms, they were assigned identification numbers and completed an anonymous pre-survey. The survey collected demographics, assessed their confidence level in tourniquet application skills, and noted any previous experience they had with VR. Standard hemorrhage control training was conducted by an EMT instructor, running 30 minutes in length, as per their established curriculum. This course included hands-on training that reviewed how to apply PPE, assess victims, identify the location of life-threatening bleeding, apply pressure while packing a wound, and/or apply a tourniquet. Proper tourniquet technique and correct tourniquet application sequence were taught as well. The participants were each given tourniquets to practice alongside the instructor, which is a standard for the course. Following training, an initial skills assessment was conducted for baseline data using the NYS EMT Certification of Skills rubric [15].

Intervention 

Thirty-five days following the initial assessment, only the VR group participated in a tourniquet placement refresher using the VR program. The 35-day period was chosen due to the duration of the EMT course length of three months. The VR program was created by TrainXR, LLC (Las Vegas, NV) and entailed being shown skills by a virtual instructor, which were then replicated throughout the VR experience using an Oculus Quest 2. Although the research team did help guide the development of the program, there are no financial/personal conflicts of interest present. The program’s goal is to utilize its sequence-driven learning aspects in order to teach tourniquet application and improve retention through its realistic environment. The VR program consists of three modes, (1) tutorial, (2) training, and (3) testing. The participants in the intervention group trained on day 35 using all 3 modes. The tutorial mode allowed the trainee to follow a virtual instructor step-by-step for the tourniquet application. The training mode provided an opportunity for repeat practice without the virtual instructor and had prompts when mistakes were made. Finally, the testing mode created stress inoculation and an emergent environment requiring the participant to place a tourniquet in a time-sensitive manner (under 90 seconds) while following all of the correct steps. The stress inoculation environment and time-sensitive manner were created to simulate a real trauma situation. A video of the VR program can be seen in Video 1. The logistics and setup of the VR intervention were organized and set up by high school and college students without formal training in hemorrhage control, purposefully done to avoid any confounding by having medical professionals present. The control group had no intervention period as they provided baseline data about the retention of tourniquet skills without having a VR refresher. 

**Video 1:** VR Tourniquet Training. The VR training includes three distinct modes: Instructor Training, Hands-On Training, and Real-World Training VR: Virtual Reality

Final assessment

Seventy days post-initial assessment, all participants were invited to participate in a final assessment. Printed versions of the same NYS EMT certification of skills rubric were used to evaluate the EMT students. Critical steps, which resulted in failure if missed included correctly applying PPE, applying the tourniquet in the correct location, tightening the tourniquet adequately, and verbalizing when the bleeding had stopped [15]. Finally, participants completed a post-survey regarding reactions to the program, complaints, and feedback. 

Raters and skills assessment

Due to the nature of the intervention, participants were not blinded, but outcome rates were. The raters were emergency medicine (EM) physicians and EMT instructors, blinded to the study protocol and interventions. Raters were calibrated using a standardized scoring checklist from the NYS EMT certification of skills rubric and assessed through ratings of mock tourniquet application tests. After both the initial and final phases, raters scored students on tourniquet skills by utilizing a printed copy of the NYS EMT Tourniquet Skills Rubric as an assessment tool. The testing site was a room that contained a Combat Application Tourniquet (CAT), and a Laerdal SimMan 3G bleeding module accessory: an arm with an arterial bleed. An active shooter scenario was read to participants, and they were then observed and scored based on their tourniquet application skills, as well as time to placement. Raters included specific errors made by the participants if they completed the task incorrectly. 

Statistical analysis 

All statistical analyses were conducted using R statistical software version 4.0.2 [16]. The primary outcome of interest was successful tourniquet placement between the VR and control groups. This was evaluated using a binomial logistic regression while controlling for previous tourniquet experience, age, gender, and class attended. Since there were multiple classes in the study, possible differences between each group were controlled for in the analysis. Secondary outcomes detailing causes of error in tourniquet application such as tightness, correct location, the correct order of steps, verbalizing steps, and utilizing gloves were similarly evaluated with a binomial logistic regression. Continuous data, which included the number of total errors and time to apply a tourniquet were evaluated using linear regressions and controlled for the same variables. All survey data was evaluated using ordinal logistic regressions. Variance inflation factors were used to check for multicollinearity for all regressions. For linear and logistic regressions, influential values were inspected using Cook’s distance, and linearity was visually inspected for age as an independent variable. The existence of proportional odds was evaluated using the Brant test for all ordinal logistic regressions. These analyses were then repeated to compare performance before and after each individual intervention. Reasons for failure were then examined using a chi-squared test. Finally, a one-way ANOVA was done to evaluate the difference in the total number of errors during the final assessment between the three different classes in the study.

## Results

The study was conducted over the course of 1 semester. Forty participants provided informed consent, were randomized, and assessed: 19 (47%) in the control group and 21 (53%) in the VR group. Participants were on average 22.5 years old (SD = 5) and male (68%, n = 27). Most participants had no previous tourniquet experience (60%, n = 24). Class 1 had n = 10, Class 2 had n = 15, and Class 3 had n = 15, with all split into the two study arms (Table 1). Between both the control and VR groups, there was no significant difference in age (22.0 vs 23.0, p = 0.52), gender (p = 0.97), and prior tourniquet experience (0.41 vs 0.33, p = 0.73) respectively.

**Table 1:** List of descriptive data in control and virtual reality study groups. VR: Virtual Reality

Characteristic	Control Group (n = 19)	VR Group (n = 21)
Age, mean	22.0 (1.0, 26.0)	23.0 (21.0, 26.0)
Male Sex	12 (71%)	15 (71%)
Prior Experience	7 (41%)	8 (38%)
Class Number		
Class 1	5 (26%)	5 (24%)
Class 2	7 (37%)	8 (38%)
Class 3	7 (37%)	8 (38%)

Skill assessment: overall performance

**Figure 2:**
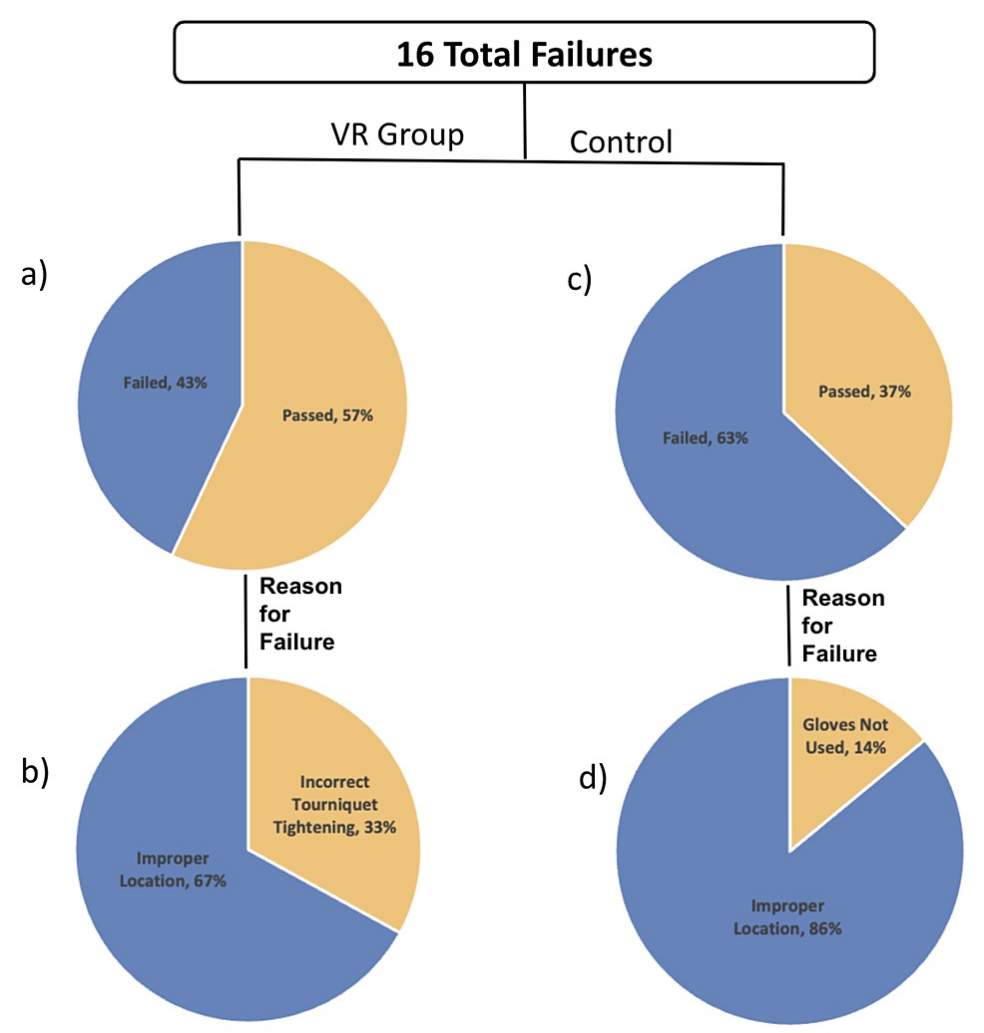
Tourniquet placement comparison of the intervention (VR) and control (no VR) group. (a) Tourniquet placement failure percentage of VR participants; (b) Tourniquet placement failure percentage of control participants; (c) Reasons for failure among VR participants who failed; (d) Reasons for failure among control participants who failed. VR: Virtual Reality

There was no significant difference in the initial correct tourniquet skill performance (89% vs 76%, p = 0.42) and the final correct tourniquet skill performance in training between the control and VR groups (63% vs 57%, p = 0.57) respectively. There was also no significant difference in the average amount of errors made during the initial assessment (0.11 vs 0.24, p = 0.30) and during the final skills assessment (1 vs 0.85, p = 0.81) between the control and VR groups respectively. In the VR group, 43% (9/21) of participants failed to apply the tourniquet correctly. In examining the causes of failure for these participants, improper tightening of the tourniquet (n = 6) or improper location (n = 3) were the errors found. In the control group, 37% (7/19) of participants failed to apply the tourniquet correctly (n = 7). None of the control participants failed due to incorrect tourniquet tightening, but rather due to improper location (n = 6) or failure to wear gloves (n = 1) (Figures 2a-2d).

**Figure 3:**
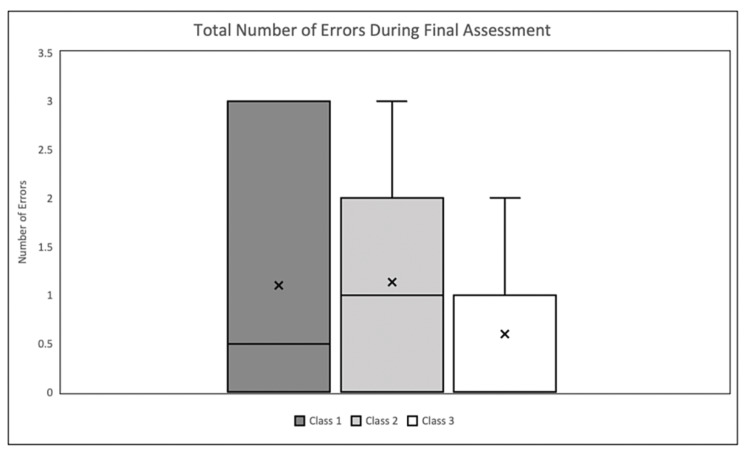
Total amount of errors for each of the three classes during the final assessment.

Additionally, it was found that those who participated in a VR session were 12.27 times more likely to fail due to improper tightening during the final assessment as compared to their initial assessment (p = 0.04) (Table 2). While the VR program did not provide any additional retention benefit in this pilot study, it did demonstrate effectiveness in aiding the retention of PPE donning during the process of tourniquet application. According to the binomial logistic regression, the VR group participants were 8.39 times more likely to apply gloves during tourniquet application in the final assessment compared to the initial assessment (p = 0.01) This increase in PPE use was not apparent in the control group regression (2.90, p = 0.16). Finally, there was no significant difference in the number of total errors during the final assessment between each class (Class 1 = 1.1, Class 2 = 1.13, Class 3 = 0.6, p = 0.28) (Figure 3).

**Table 2:** Binomial Logistical Regression Analysis of Dependent Variables for VR and Control Groups. The VR group was significantly more likely to have an error associated with incorrect tightening and was significantly more likely to use gloves. *statistically significant VR: Virtual Reality

Initial Assessment vs. Final Assessment				
Intervention	No VR		VR	
Dependent Variable	Odds Ratio or Estimate	P-value	Odds Ratio or Estimate	P-value
Applied Tourniquet Correctly	0.12	0.07	0.39	0.18
Tourniquet Applied in Correct Sequence	0.00	1.00	0.33	0.31
Tourniquet at Correct Location	0.12	0.07	0.70	0.67
Incorrect Tightening of Tourniquet	1.00	1.00	12.27	*0.04
Utilized Gloves	2.90	0.16	8.39	*0.01
Verbalized Bleeding Stopped	1.00	1.00	0.00	1.00

Intervention group perceptions on VR training

**Figure 4:**
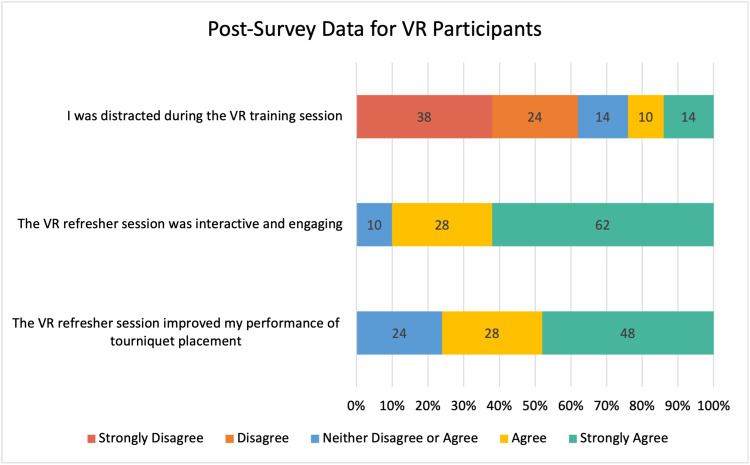
Post-survey results regarding perceptions, comfort, and feedback for the VR participants in the study. VR: Virtual Reality

The participants in the VR group reported feeling more comfortable and confident handling tourniquets and hemorrhagic emergencies after using the VR trainer. According to survey results, on a 5-point Likert scale, (1 strongly disagree and 5 strongly agree), 76% (16/21) of the participants either strongly agreed or agreed with the statement that utilization of the VR trainer improved their performance. Additionally, 90% of the participants (19/21) either strongly agreed or agreed with the statement that the VR program was interactive and engaging. Lastly, 62% of the participants (13/21) reported not being distracted at all during the program or only experiencing minor distractions during the immersive experience (Figure 4).

## Discussion

This study sought to examine the added benefit of an immersive VR hemorrhage control training experience on tourniquet performance skills in EMT students. No significant benefit was identified in correct tourniquet placement performance compared to a standard EMT hemorrhage control training program (63% vs 57%, p = 0.57). Importantly, adding VR was associated with failures primarily due to incorrect tourniquet tightening while the control group failed due to errors related to steps of the procedure (e.g. improper location of tourniquet). Errors within the VR group may be due to the haptics limitations of VR. Haptics is important in medical training as it simulates real life by allowing the users to touch and feel objects that they interact with [17]. Research has found that the use of valid haptics can improve skill performance and can decrease the chance of making errors in medical procedures [18,19]. However, Wang et al. discovered that haptic sensations with VR are poor and do not compare to the sensations obtained through physical touch [17]. As a result, the poor haptics within the VR program may account for a portion of the errors made by the VR group as the technology is unable to simulate the extent of tightening required when using a real tourniquet.

This indicates a limitation of VR: while it is capable of enabling students to practice procedure-based skills in a simulated environment, haptic deficiencies limit the immersive experience and potentially impact the ability to correctly perform certain procedures. On the other hand, the control group’s errors were not haptic-related but seem to be more related to tourniquet placement and other details that were adequately taught by the VR refresher. Therefore, the control group’s errors may be correlated to the lack of a refresher in medical training, as various studies have found that refreshers are able to increase retention, performance, and comfort of medical skills [20,21]. Since the VR group had an intervention before the final assessment, it may have led them to make non-haptic errors comparatively. The addition of a refresher course, as well as VR’s immersive training benefits, likely explains the VR participants' feelings of increased comfort and confidence with tourniquet placement after using the VR experience.

The results found that the addition of VR training had no significant difference in skill performance of hemorrhage control skills. The research team has investigated a few avenues to improve the retention of hemorrhage control skills. Dadario et al. tested the use of a visual-based mobile application on tourniquet placement and found that participants who used the app had significantly better tourniquet situational awareness skills (e.g., correct tourniquet placement) than the non-application users [8]. Jafri et al. used simulation for basic tourniquet training and found improvement in short-term skills but not in retention after three months [7]. VR may be an alternative modality that can be useful in both improving tourniquet skills and retention due to its immersive and asynchronous nature, along with its ability to simulate realistic scenarios in medical training [22]. Literature has found that VR can lead to improvement in procedural skills for different groups of healthcare personnel. Kravitz et al. found that the use of a VR trainer improved (PPE) donning and doffing among medical residents [9], while Moll-Khosrawi et al. found that the use of VR significantly enhanced Basic Life Support (BLS) training among medical students [23]. While this study did not find a difference between control and intervention groups, the fact that the majority of VR-group errors were related to haptics, lends the possibility that an improvement in design and technology may mitigate this current deficiency in VR technology and further advance the ability to train and retain tourniquet skills through an immersive, virtual format.

Limitations

The current study recruited from a heterogeneous convenience sample of participants signed up for an EMT course. Given the recent pandemic, this study was underpowered due to low enrollment in these EMT classes. This may have further reduced the ability to detect meaningful intervention effects. Additionally, inter-rater reliability was not controlled for during the initial and final assessment since there were different blinded raters for each class. Lastly, the retention period was a limitation of the study, as the 70-day retention period was the longest time period attainable accounting for the length of a singular semester and allowing time for students to prepare for their actual EMT certification exams. Ideally, a retention period of six months should be used to see how skills would naturally fade within a longer span of time [24].

## Conclusions

In this study, there was no significant skill difference between control and VR intervention groups. However, the VR group was more likely to make errors due to incorrect tourniquet tightening compared to the control group. Additionally, the VR group was less likely to make procedure-related errors (e.g., not utilizing gloves) than the control group. There is a need for improved haptics simulation during VR training for tourniquet placement. Further research recommendations include comparing VR to in-person training as a refresher, improving haptic feedback during VR experiences, and testing asynchronous training by distributing VR headsets rather than testing them with an observer, which is likely how this training will be implemented in the near future. 
